# Tumor-targeted bioactive nanoprobes visualizing of hydrogen peroxide for forecasting chemotherapy-exacerbated malignant prognosis

**DOI:** 10.3389/fbioe.2023.1226680

**Published:** 2023-08-11

**Authors:** Fan Zhang, Yong Jia, Fangman Chen, Yawei Zhao, Li Li, Zhimin Chang

**Affiliations:** ^1^ CAS Key Laboratory of Bio Medical Diagnostics, Suzhou Institute of Biomedical Engineering and Technology Chinese Academy of Sciences, Suzhou, China; ^2^ Zhengzhou Institute of Biomedical Engineering and Technology, Zhengzhou, China; ^3^ School of Nursing, Jilin University, Changchun, Jilin, China

**Keywords:** bioactive, nanoprobe, cancer, hydrogen peroxide, chemotherapy

## Abstract

**Introduction:** Fluorescent visualization of hydrogen peroxide in the tumor microenvironment (TME) is conducive to predicting malignant prognosis after chemotherapy. Two photon microscopy has been employed for *in vivo* hydrogen peroxide detection owing to its advantages of deep penetration and low phototoxicity.

**Methods:** In this study, a two-photon fluorescent probe (TPFP) was protected by mesoporous silica nanoparticles (MSNs) and masked by cloaking the cancer cell membranes (CM), forming a tumor-targeted bioactive nanoprobe, termed MSN@TPFP@CM.

**Results:** This multifunctional nanoprobe allowed for the effective and selective detection of excessive hydrogen peroxide production in chemotherapeutic Etoposide (VP-16)-challenged tumor cells using two-photon microscopy. After specific accumulation in tumors, VP-16-MSN@TPFP@CM monitored tumor-specific hydrogen peroxide levels and revealed a positive correlation between oxidative stress in the TME and chemotherapy-exacerbated malignant prognosis.

**Discussion:** Given the recent translation of fluorescent imaging into early clinical trials and the high biocompatibility of bioactive nanoprobes, our approach may pave the way for specific imaging of oxidative stress in solid tumors after treatment and provide a promising technology for malignant prognosis predictions.

## 1 Introduction

Reactive oxygen species (ROS) are considered secondary messengers in biological organisms and are critical for the regulation of pathological and physiological processes, such as cell growth and differentiation, immune response, and aging (Circu and Aw, 2010; Liou and Storz, 2010; Schieber and Chandel, 2014). Mounting evidence suggest that continuous production, transformation, and consumption of ROS can promote pro-survival and pro-proliferative pathways, and metabolic adaptation of tumor cells to the tumor microenvironment (TME) (Jensen, 1966; Halliwell et al., 2000; Gupta and Massagué, 2006; Marcu, 2014; Liu et al., 2023). Recently, aberrant production of ROS in the TME has been associated with cancer malignant prognosis, especially in chemotherapy-exacerbated malignant prognosis (Lambert et al., 2017; Jiang et al., 2020). Under such conditions, sublethal levels of ROS induced by chemotherapeutics can help propagate, amplify, and effectively create a mutagenic and oncogenic field that facilitates tumor repopulation and acts as a springboard for metastatic tumor cells (Saggar and Tannock, 2015; Verma et al., 2016; Duy et al., 2021). Therefore, monitoring ROS levels in the TME is conducive for understanding cancer progression and developing novel therapeutics.

As the most stable ROS, hydrogen peroxide (H_2_O_2_) has a lifetime of up to a few minutes and can diffuse across biological membranes, thereby functioning as an ideal biomarker for cancer progression (Kamata et al., 2005; Lin and Beal, 2006; Guo et al., 2014; Jung et al., 2016). Electrochemistry and luminescence are the two major strategies for the quantification of H_2_O_2_ in living systems (Wang et al., 2022). The former measures extracellular H_2_O_2_ in an invasive manner and is affected by biofouling (Song et al., 2010; Aghamiri et al., 2019; Zhao et al., 2019). Whereas, the latter method is suitable for extracellular and intracellular H_2_O_2_ with the advantages of noninvasiveness, simple operation, high sensitivity, and excellent spatiotemporal resolution (Lu et al., 2021; Zhou et al., 2021; Hao et al., 2022; Zhan et al., 2022). To overcome limited light penetration depth, two-photon microscopy (TPM) has been employed for H_2_O_2_ detection with less phototoxicity and lower self-absorption, which facilitates real-time measurements *in vivo* (Chen et al., 2013; Guo et al., 2013; Li et al., 2017; Shi et al., 2018; Liaw et al., 2021). Additionally, as a benefit of nanotechnology, nanoparticulate probes have been widely developed for the efficient imaging of H_2_O_2_ in cancer owing to their specific tumoral targeting, higher penetration, and good stability (Maji et al., 2018; Shao et al., 2018; Wang et al., 2019; Zhang et al., 2019; Bondon et al., 2022). Therefore, it is necessary to develop a novel two-photon fluorescent nanoprobe with near-infrared (NIR) emission to monitor H_2_O_2_ during cancer chemotherapy.

**SCHEME 1:**
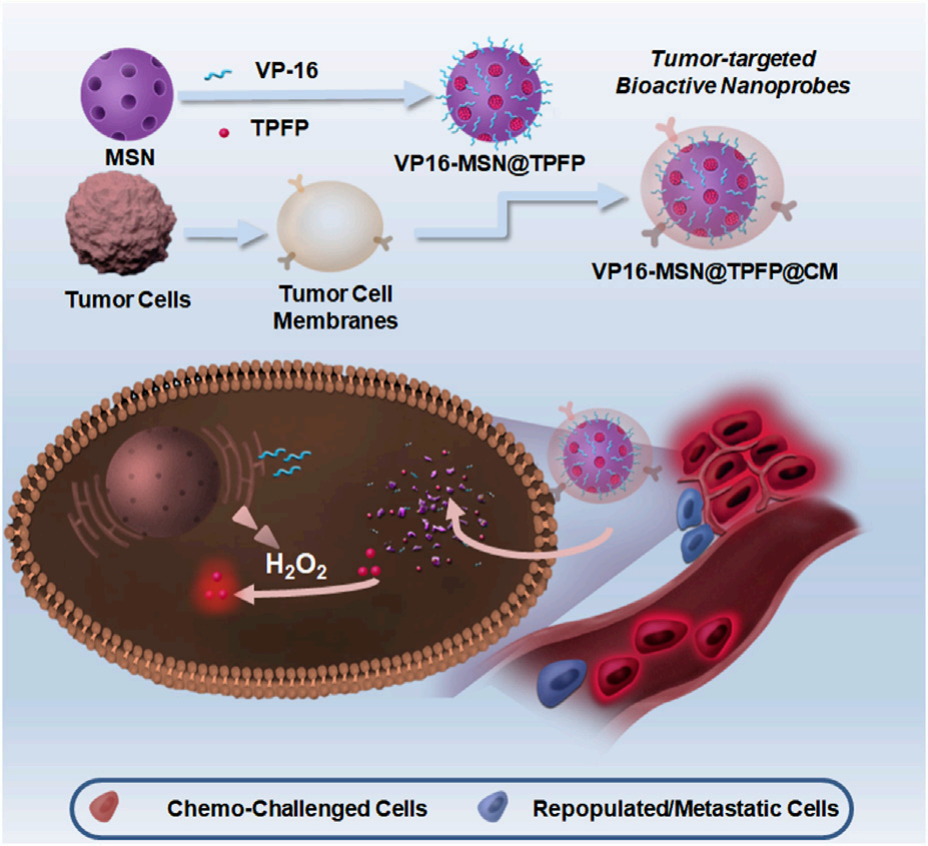
Schematic illustration of synthesis of VP-16-MSN@TPFP@CMs and their applications in visualizing hydrogen peroxide (H_2_O_2_) for forecasting chemotherapy-exacerbated malignant prognosis.

In this study, we created a tumor-targeting bioactive nanoprobe which facilitated two-photon fluorescence imaging to visualize H_2_O_2_ during cancer chemotherapy (Scheme 1). We incorporated an H_2_O_2_-responsive two-photon fluorescent probe (TPFP) into mesoporous silica nanoparticles (MSNs), which were then coated with cancer cell membranes to form bioactive nanoprobes named MSN@TPFP@CMs. After loading with chemotherapeutic Etoposide (VP-16), this multifunctional nanoprobe allowed the effective and selective detection of excessive H_2_O_2_ production in chemotherapy-challenged tumor cells through two-photon microscopy. Importantly, VP-16-MSN@TPFP@CMs preferably accumulated in tumors and monitored tumor-specific H_2_O_2_ levels in a subcutaneous breast cancer mouse model without immediate or delayed toxic effect, revealing a positive correlation between endogenous H_2_O_2_ in the TME and chemotherapy-exacerbated repopulation and metastasis *in vitro* and *in vivo*. Two-photon fluorescence detection of H_2_O_2_ in the TME may be an appealing strategy for predicting poor prognosis after cancer chemotherapy, including recurrence and metastasis.

## 2 Materials and methods

### 2.1 Fabrication of VP-16-MSN@TPFP@CMs

A solution of the H_2_O_2_-responsive probe TPFP was prepared based on previous studies. MSNs were synthesized as follows: 0.12 g cetrimonium tosylate (CTAT), 0.03 g triethanolamine (TEAH_3_), and 10 mL deionized water were mixed and stirred at 80°C for 30 min. Subsequently, a solution of 1.0 g tetraethyl orthosilicate (TEO) was added dropwise to the surfactant solution. The resulting mixture was stirred at 80°C for another 4 h at 1,000 rpm. The products were collected by centrifugation (10,000 rpm 30 min), washed three times with ethanol, and subsequently refluxed in an ethanol solution of NH_4_NO_3_ (1% w/v) for 12 h. Briefly, we used VP-16 and TPFP (1:20 mass ratio) dissolved in DMSO for the co-loading of the probes. To configure probes containing different ratios of chemotherapeutic components, the mass ratio of VP-16 to TPFP was adjusted to 0.5:20, 1:20, and 2:20 to prepare VP-16-MSN@TPFP (L), VP-16-MSN@TPFP (M), and VP-16-MSN@TPFP (H). The nanoparticles were collected by high-speed centrifugation and resuspended in the aqueous phase. Subsequently, VP-16-MSN@TPFP was mixed with CM derived from 4T1 cells, sonicated with heating for 5 min, and subsequently extruded through 200 nm polycarbonate membranes to obtain VP-16-MSN@TPFP@CMs.

### 2.2 Characterization of VP-16-MSN@TPFP@CMs

The morphology of the MSNs was characterized using a JEM-2100F transmission electron microscope (TEM; JEOL, Ltd., Japan) and a scanning electron microscope (SEM; FEI Quanta 200F). The hydrodynamic diameter and zeta potential of the nanoparticles in water and PBS were characterized using a Nano-ZS 90 Nanosizer (Malvern Instruments Ltd., Worcestershire, United Kingdom). UV-vis absorption spectra were recorded using a U-3310 spectrophotometer (Hitachi, Japan). Sodium dodecyl sulfate-polyacrylamide gel electrophoresis (SDS-PAGE) was used to characterize the protein composition of nanoparticles. Stability experiments were performed by measuring the nanoprobes in Dulbecco’s Modified Eagle’s medium (DMEM) plus 10% FBS for 7 days using dynamic light scattering (DLS).

### 2.3 Cell culture and *in vitro* analysis

The mouse breast cancer 4T1 cell line was cultured in DMEM supplemented with 10% FBS along with penicillin and streptomycin at 100–100 U/mL, respectively. Cells were incubated at 37°C in 5% CO_2_-95% air atmosphere.

For the detection of exogenous H_2_O_2_, 4T1 cells were incubated with nanoprobes at 37°C for 30 min, images of VP-16-MSN@TPFP@CMs in live cells were investigated via spectral confocal multiphoton microscopy (Olympus FV1000-IX81) with a high-performance model titanium-sapphire laser source (Maitai, Spectra-Physics, United States), with the emission ranging from 575 to 630 nm. The excitation wavelength was 860 nm with a constant intensity.

#### 2.3.1 Chemo-malignant prognosis cell model

Single-cell suspensions of chemotherapy-challenged cells were collected using trypsin after 12 h of treatment with VP-16 (2.5 μg/mL). The chemotherapy-challenged cells were re-inoculated in 6-well plates at a density of 5 × 10^4^/well, and the cells were collected after 24 h to obtain whole cell protein lysates. After determining the protein concentration of the samples using the bicinchoninic acid (BCA)method, the Cyclooxygenase 2 (COX2) content in each treatment group was measured using a COX2 ELISA assay kit (ab210574). Additionally, the culture medium supernatant was collected for the determination of Prostaglandin E2 (PGE2) using an ELISA kit (ab287802).

#### 2.3.2 Measurement of tumor cell repopulation with bioluminescence imaging

We constructed a chemo-repopulation cell model based on the fact that the luciferase activity of Fluc-labeled 4T1 cells was tightly correlated with cell number. The 4T1-Fluc cells (100 cells) were seeded with differentially agents-treated 4T1 cells (1 × 10^4^) in 24-well plates. During the co-culture period (12–14 days), the culture medium was replaced with fresh 5% FBS DMEM every 3 days. Finally, to measure the luciferase activity of 4T1-Fluc, 0.15 mg/mL D-Luciferin potassium in PBS was added to each well before bioluminescence imaging.

#### 2.3.3 Measurement of tumor cell metastasis with transwell assay

For the transwell assay, 4T1 cells were seeded into up-chamber of 8 μm pore size in six-well plates. Subsequently, 1.5 mL chemo-challenged 4T1 cell (5 × 10^4^) medium was added into the lower chamber of every well and 500 μL serum free DMEM containing 4T1 cells (3 × 10^4^) was added into the up-chamber. After 20–24 h, 4T1 cells remaining in the inserts were gently removed using cotton swabs. Migratory 4T1s were fixed in 90% ethanol and stained with crystal violet. The number of migratory 4T1s was measured by counting the cells from five random fields under a microscope.

#### 2.3.4 Correlation analysis

The mean fluorescence intensity from flow cytometry of H_2_O_2_ levels in chemotherapy-challenged cells, PEG2 levels, and COX2 levels were plotted to analyze the correlation between H_2_O_2_ and chemo-repopulation/metastatic cells.

### 2.4 Animals and *in vivo* analysis

#### 2.4.1 Chemo-malignant prognosis in 4T1 mouse model

All animal experimental procedures were approved by the Ethics Committee for the Use of Experimental Animals of the Suzhou Institute of Biomedical Engineering and Technology of the Chinese Academy of Science (Suzhou, Jiangsu, China). Initially, Balb/C NuNu approximately 18 g each) female mice (*n* = 8) aged four–six weeks were obtained from Cavens Biogle (suzhou) Model Animal Research Co., Ltd. A xenograft tumor model was established by subcutaneous injection of 1 × 10^6^ 4T1 cells into the right mammary fat pads.

When the tumor size reached approximately 150 mm^3^, the nanoprobes (0.1 mg/kg based on VP-16) were administered intravenously. For the chemo-malignant prognosis in the 4T1 mouse model, tumor volumes and body weights were recorded every alternate day after the first injection. Mice were sacrificed 14 days after chemotherapeutic stimulation and lung tissue was collected to count the number of pulmonary metastatic nodules. Additionally, 12 h after the intravenous injection of nanoprobes, the tumor site fluorescence signal was monitored using *In-Vivo* Xtreme II™. Subsequently, the number of pulmonary metastatic nodules versus the *in vivo* fluorescence signal of the TPFP was plotted to analyze the correlation between chemo-malignant prognosis and H_2_O_2_ levels.

#### 2.4.2 *In vivo* biodistribution and biosafety

The 4T1 tumor-bearing mice were injected with VP-16-MSN@TPFP or VP-16-MSN@TPFP@CM solutions (2 mg/kg based on TPFP). The fluorescence intensity of the TPFP in each organ sample was measured. The fluorescence intensity was converted to TPFP mass to investigate the organ distribution 12 h after administration. After treatment for 14 days, the mice were sacrificed, and the main organs (liver, spleen, kidneys, heart, and lungs) were collected for hematoxylin and eosin (H&E) stain to analyze the pathophysiology. Biochemical parameter indices were also tested for acute toxicological assay.

### 2.5 Statistical analysis

Student’s *t*-test was used to analyze the differences between two groups. Differences between more than two groups were analyzed using a one-way analysis of variance. Simple linear regression was used to analyze the correlation between H_2_O_2_ signals and the expression level of COX2, PEG2 and their ratios. Sample sizes (n) and *p*-values (p) for all statistical analyses are indicated in figures and figure legends. Data were analyzed using statistical software OriginPro 2021b. In all cases, *p* < 0.05 represents a statistically significant difference.

## 3 Results and discussion

### 3.1 Preparation and characterization of VP-16-MSN@TPFP@CMs

**FIGURE 1:**
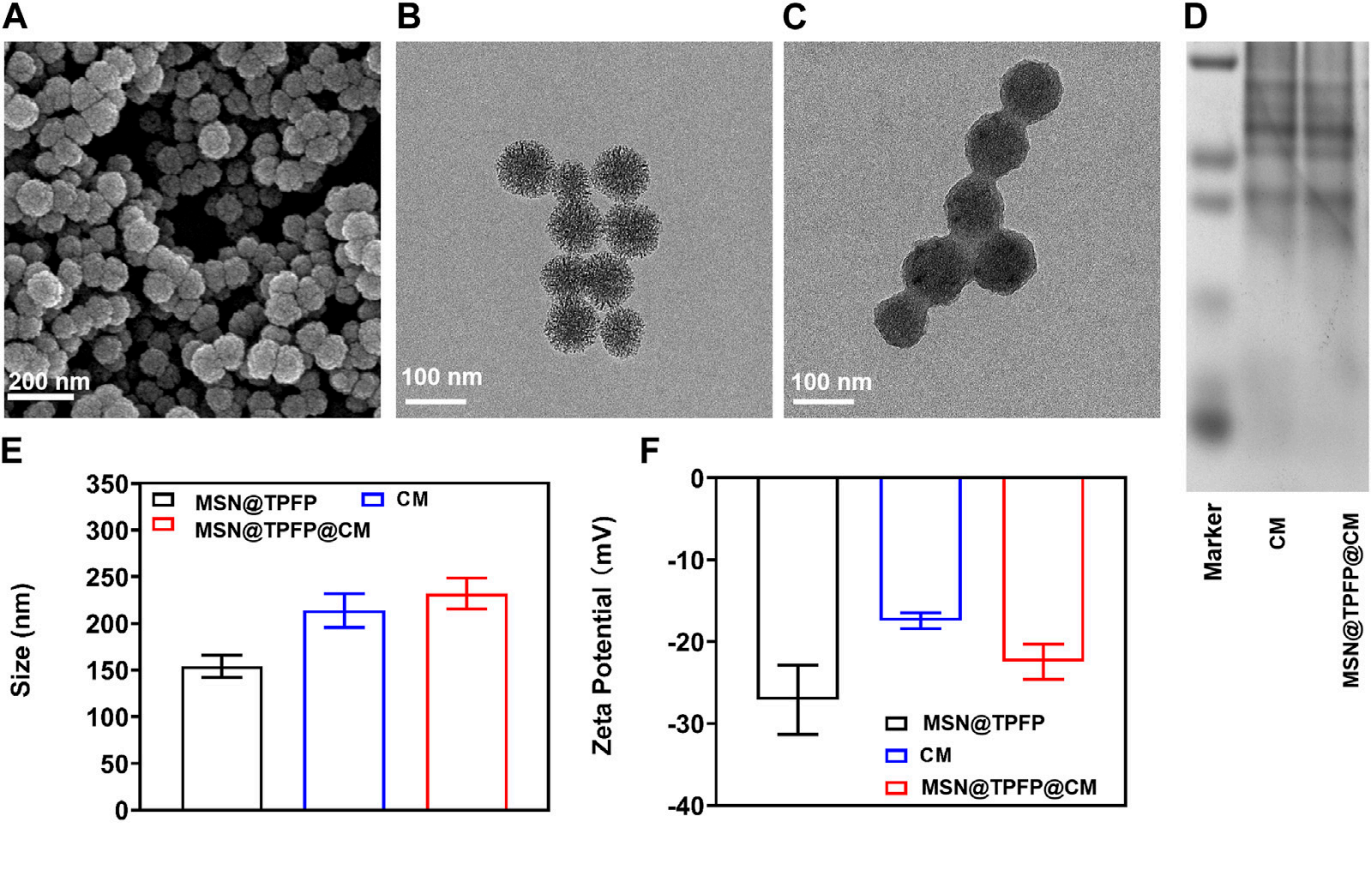
Spectroscopic studies of VP-16-MSN@TPFP@CMs. **(A)** UV-vis absorption of VP-16-MSN@TPFP@CMs. **(B)** Fluorescence spectra of VP-16-MSN@TPFP@CMs in the presence of H_2_O_2_ with excitation at 560 nm. **(C)** Fluorescence imaging of 4T1 cells incubated with TPFP or VP-16-MSN@TPFP@CMs for 30 min. Particle size **(D)**, Zeta potential **(E)**, and SDS-PAGE results **(F)** of VP16-MSN@TPFP@CM.

Mesoporous silica materials (MSNs) were prepared as previously reported. The SEM and TEM images revealed monodisperse spherical MSNs with a diameter of 80 nm (Figure 1A; Figure 1B). The prepared MSNs were loaded with the ROS-responsive fluorescent probe TPFP and chemotherapeutic VP-16 to form VP-16-MSN@TPFP. Release results show that simultaneous release of the two components can be achieved (Supplementary Figure S2). Subsequently, 4T1 breast cancer CM were isolated and coated with VP-16-MSN@TPFP to prepare VP-16-MSN@TPFP@CMs. As shown in Figure 1C, the bioactive materials possess a core–shell structure with an MSN core enclosed in a smooth membrane shell. Consistently, a slight increase in hydrodynamic diameter and a decrease in surface charge were observed after CM coating (Figure 1D; Figure 1E). Protein electrophoresis demonstrated the presence of membrane proteins on VP-16-MSN@TPFP@CMs (Figure 1F), suggesting the successful integration of the cell membrane-coated nanoplatforms. Additionally, stability experiments in 10% FBS-containing medium confirmed that VP-16-MSN@TPFP@CMs exhibited little aggregation after 7 days of incubation (Supplementary Figure S1).

### 3.2 Spectroscopic studies of VP-16-MSN@TPFP@CMs

**FIGURE 2:**
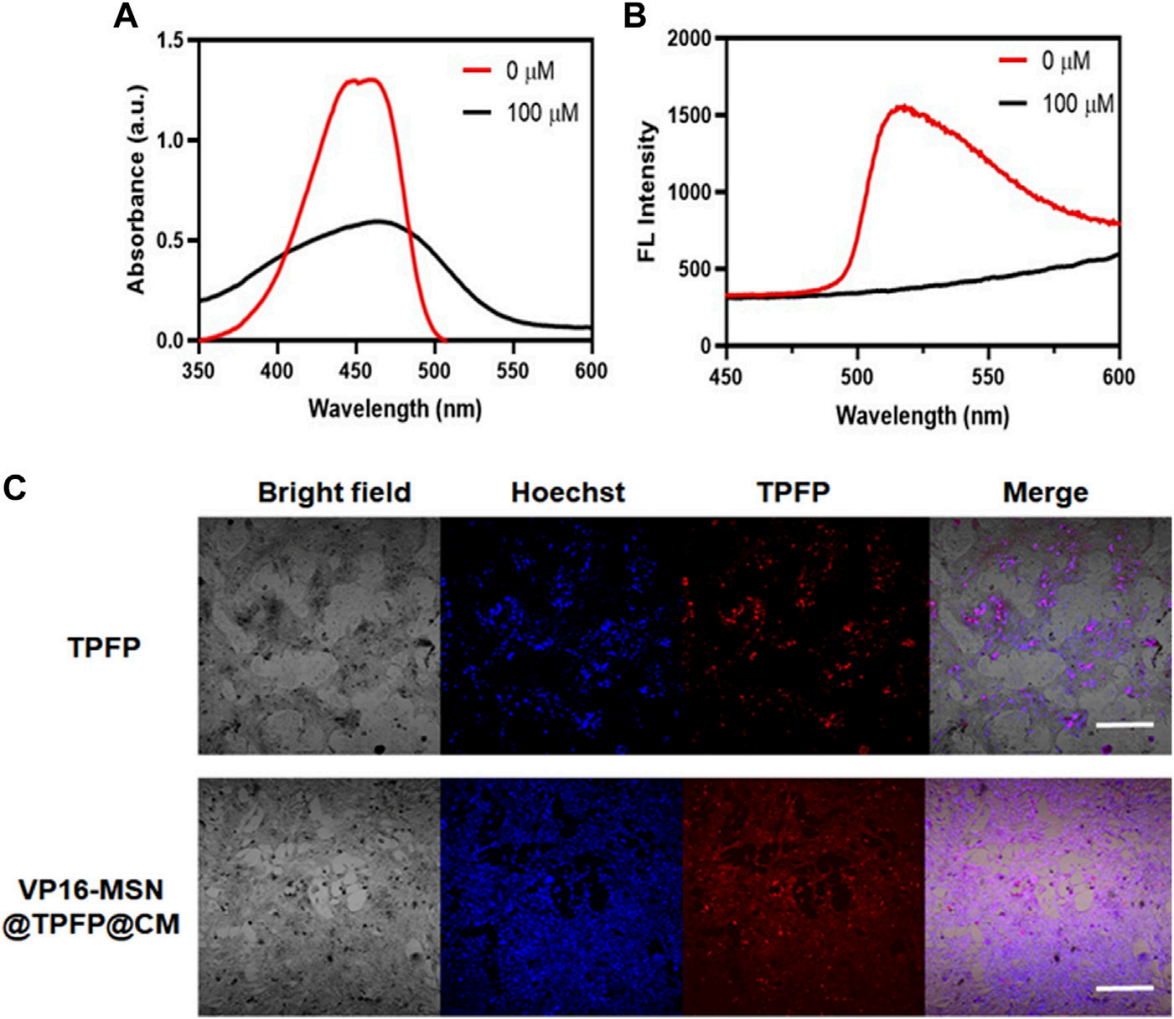
Spectroscopic studies of VP-16-MSN@TPFP@CMs. **(A)** UV-vis absorption of VP-16-MSN@TPFP@CMs. **(B)** Fluorescence spectra of VP-16-MSN@TPFP@CMs in the presence of H_2_O_2_ with excitation at 560 nm. **(C)** Fluorescence imaging of 4T1 cells incubated with TPFP or VP-16-MSN@TPFP@CMs for 30 min.

Before the *in vitro* and *in vivo* experiments, a series of optical experiments were performed to evaluate VP-16-MSN@TPFP@CM for ROS-responsive imaging properties. The absorption spectra showed a characteristic absorption of VP-16-MSN@TPFP@CM in phosphate buffer saline at 428 nm and no significant absorption at 560 nm. After adding H_2_O_2_ (100 μM) to the solution for 30 min, the absorption spectra showed a decrease at 428 nm and concomitant absorption at 560 nm (Figure 2A). In contrast, the fluorescence emission spectra showed that excitation at 560 nm after the addition of H_2_O_2_ resulted in an emission peak at 699 nm (Figure 2B). Quantitative studies have shown that the fluorescence intensity of VP-16-MSN@TPFP@CM can be used for the determination of *in vitro* and *in vivo* hydrogen peroxide concentration (Supplementary Figure S3, S4). Subsequently, we evaluated the imaging function of the prepared VP-16-MSN@TPFP@CM in 4T1 cells. As shown in Figure 2C, the fluorescence of TPFP and VP-16-MSN@TPFP@CM was found in the cytoplasm and represented H_2_O_2_ levels, indicating that VP-16-MSN@TPFP@CM was suitable for intracellular H_2_O_2_ analysis.

### 3.3 The correlation of H_2_O_2_ level in VP-16-treated cell with repopulation/metastasis *in vitro*

**FIGURE 3:**
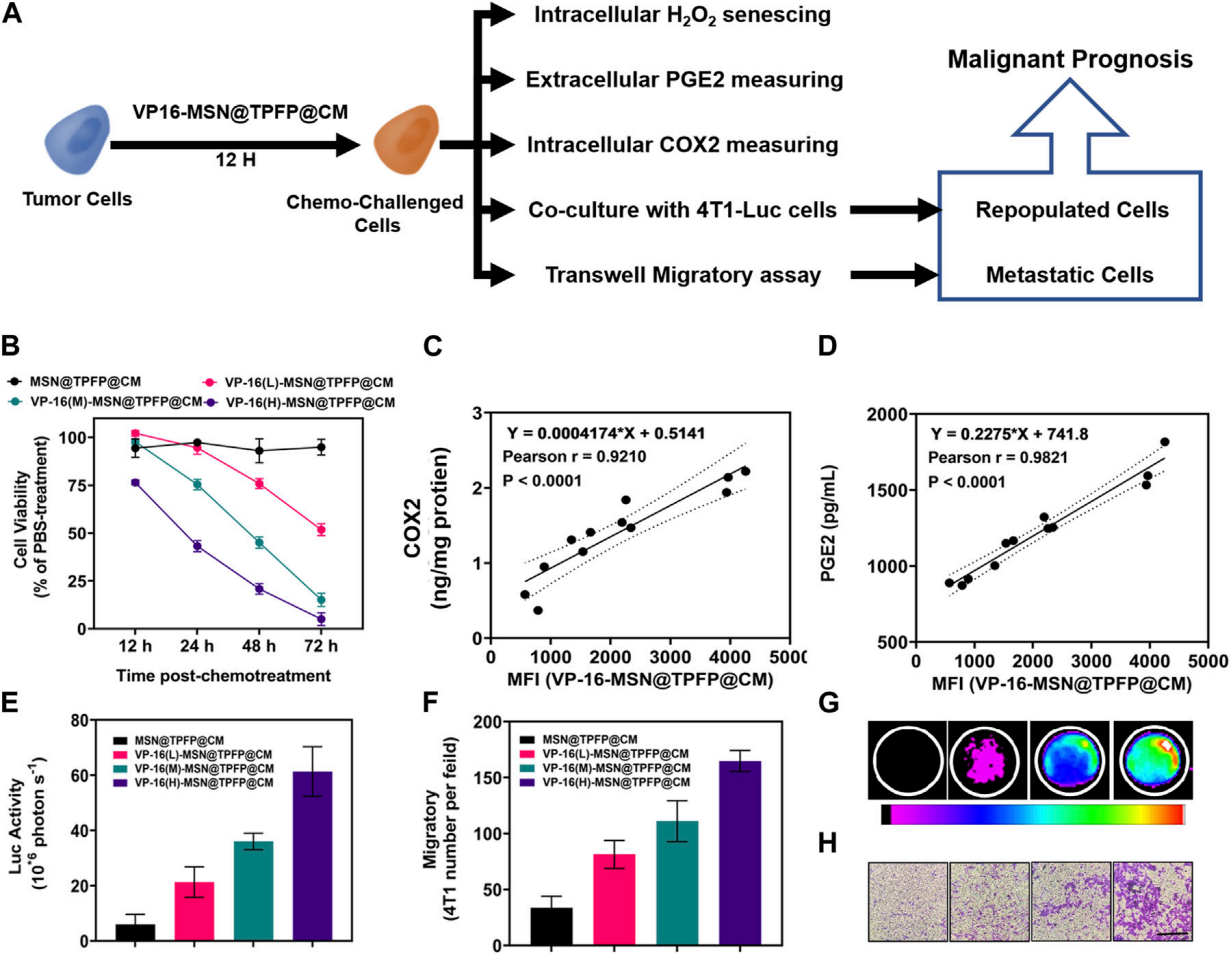
The correlation of post-chemotherapy ROS with chemo-exacerbated repopulation/metastasis cell models. **(A)** Schematic diagram. **(B)** Cell activity after treatment with different materials. The correlation analysis of ROS mean fluorescence intensity (MFI) with the expression levels of COX2 protein **(C)** or the ROS MFI with the level of PGE2 **(D)** Number of fluorescent cells in the cell repopulation model **(E,G)**. Migration rate results for each treatment group in the migration assay **(F,H)**.

To ascertain the correlation between H_2_O_2_ levels and repopulation/metastasis *in vitro* in VP-16-treated cells, we conducted the chemotherapy-exacerbated repopulation/metastasis models *in vitro* by appropriate VP-16 stimulation (Figure 3A). Cell viability results showed that co-culturing 4T1 cells in six-well plates 12 h post-chemostimulation with all three materials caused significant cell death at 72 h (Figure 3B). The mean fluorescence intensity (MFI) of the VP-16-MSN@TPFP@CMs was measured using flow cytometry 12 h after the chemotherapeutic challenge (Supplementary Figure S5). The levels of intracellular COX2 and PGE2 in the culture medium were assayed on day 2. Increased levels of both the components were observed with increasing levels of VP-16 (Supplementary Figure S6, S7). Cell repopulation and cell migration assays showed that VP-16-stimulated cells shaped a microenvironment conducive to cell repopulation and metastasis by increasing components such as the inflammatory protein COX2 and cytokine PGE2 (Figures 3E–G). The intracellular fluorescence signal after treatment with nanoprobes was measured using flow cytometry. These data are consistent with the changes in COX2 protein expression and PGE2 secretion during chemotherapy. Correlation analysis showed that the MFI of VP-16-MSN@TPFP@CMs-treated cells was highly positively correlated with COX2 expression levels (Pearson r:0.9210, *p* < 0.001) (Figure 3D) and PGE2 levels (Pearson r:0.9821, *p* < 0.001) (Figure 3C), confirming that VP-16-MSN@TPFP@CMs might provide valuable information for the evaluation of repopulation/metastasis after chemotherapy.

### 3.4 The correlation of post-chemotherapy ROS with tumor prognosis

**FIGURE 4:**
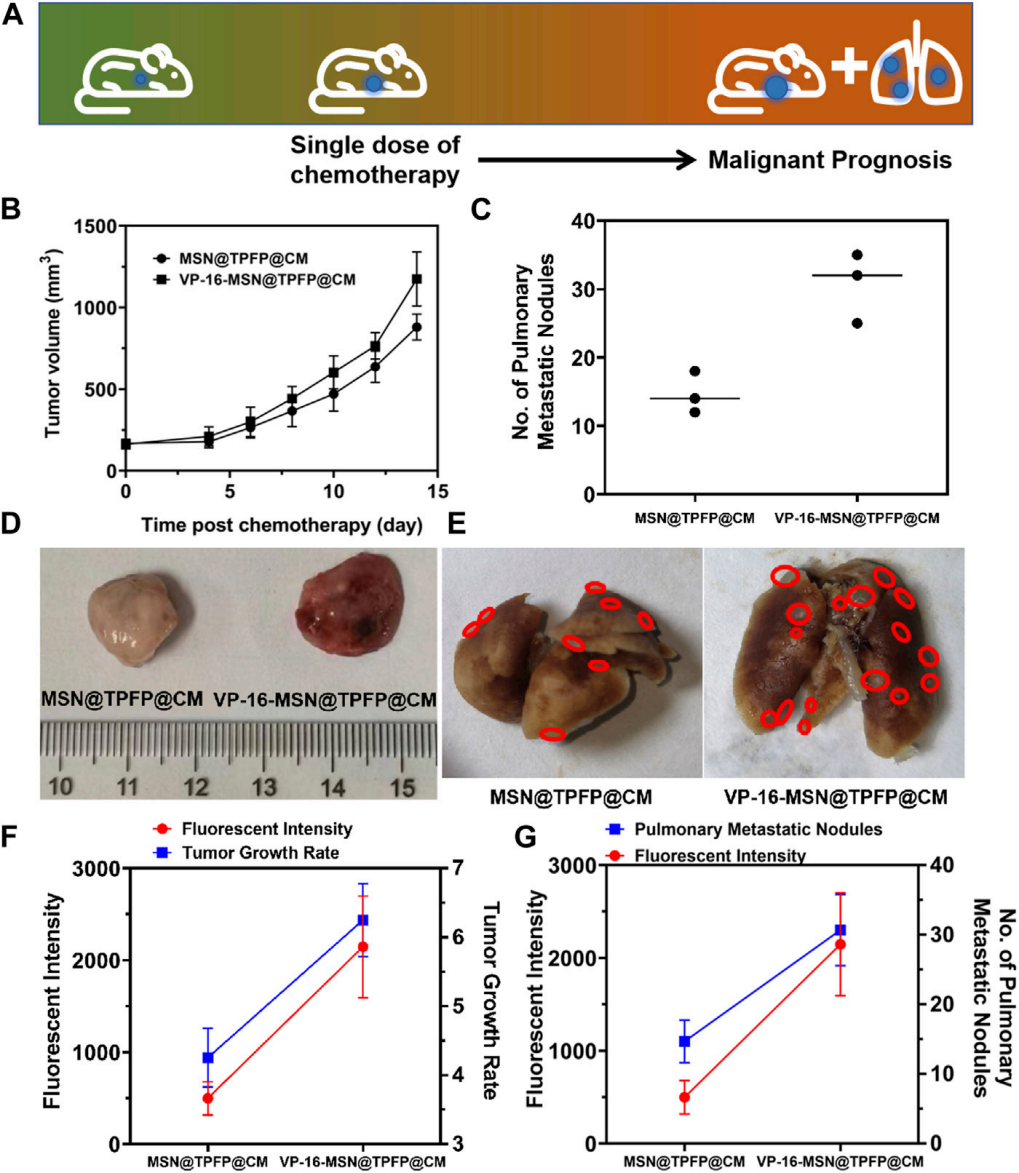
**(A)** Schematic diagram of tumor prognosis model. Orthotopic tumor growth curve **(B)** and pulmonary metastatic nodules **(C)** of chemotherapy-exacerbated model. Representative photographs of tumors **(D)** and lung tissue **(E)** from each group. The correlation analysis of the TGR(g) or pulmonary metastatic nodules **(G)** with the fluorescence intensity of orthotopic tumor.

We established a 4T1 tumor-bearing mouse model to evaluate H_2_O_2_ detection using VP-16-MSN@TPFP@CM. We examined the H_2_O_2_ signals at the tumor site after intravenous treatment with VP-16-MSN@TPFP@CM or a mixture of VP-16 and TPFP. The results showed that, compared to free small molecules, VP-16-MSN@TPFP@CM could achieve up to 12 h of H_2_O_2_ monitoring at the tumor site owing to better tumor targeting and retention (Supplementary Figure S8). To further validate the advantages of the cell membrane-cloaked nanoprobe, we compared the distribution characteristics of VP-16-MSN@TPFP and VP-16-MSN@TPFP@CM. The results showed that the cell membrane-cloaked nanoprobes accumulated more at the tumor site after 12 h of i.v. administration, thus facilitating better *in vivo* monitoring (Supplementary Figure S8). We treated the tumor-bearing mice with a single dose of VP-16-MSN@TPFP@CM (Figure 4A). At the non-therapeutic stimulation dose, the growth rate of the tumor was higher than that of cells treated with MSN@TPFP@CM, and more metastatic nodules were found (Figure 4B; Figure 4C). Subsequently, the relationship between the H_2_O_2_ levels at the tumor site port-chemo with umor growth rate (TGR) or metastatic nodules was compared between MSN@TPFP@CM and VP-16-MSN@TPFP@CM. We observed more pulmonary metastatic nodules (PMN) in the lungs of VP-16-stimulated mice, and the TGRwas higher than in mice without chemotherapy (Figure 4D; Figure 4E). Both PMN and TGR fitted well when conducting correlation analysis with the H_2_O_2_ signal of the orthotopic tumor (Figure 4F; Figure 4G).

### 3.5 *In vivo* bio-safety assays

**FIGURE 5:**
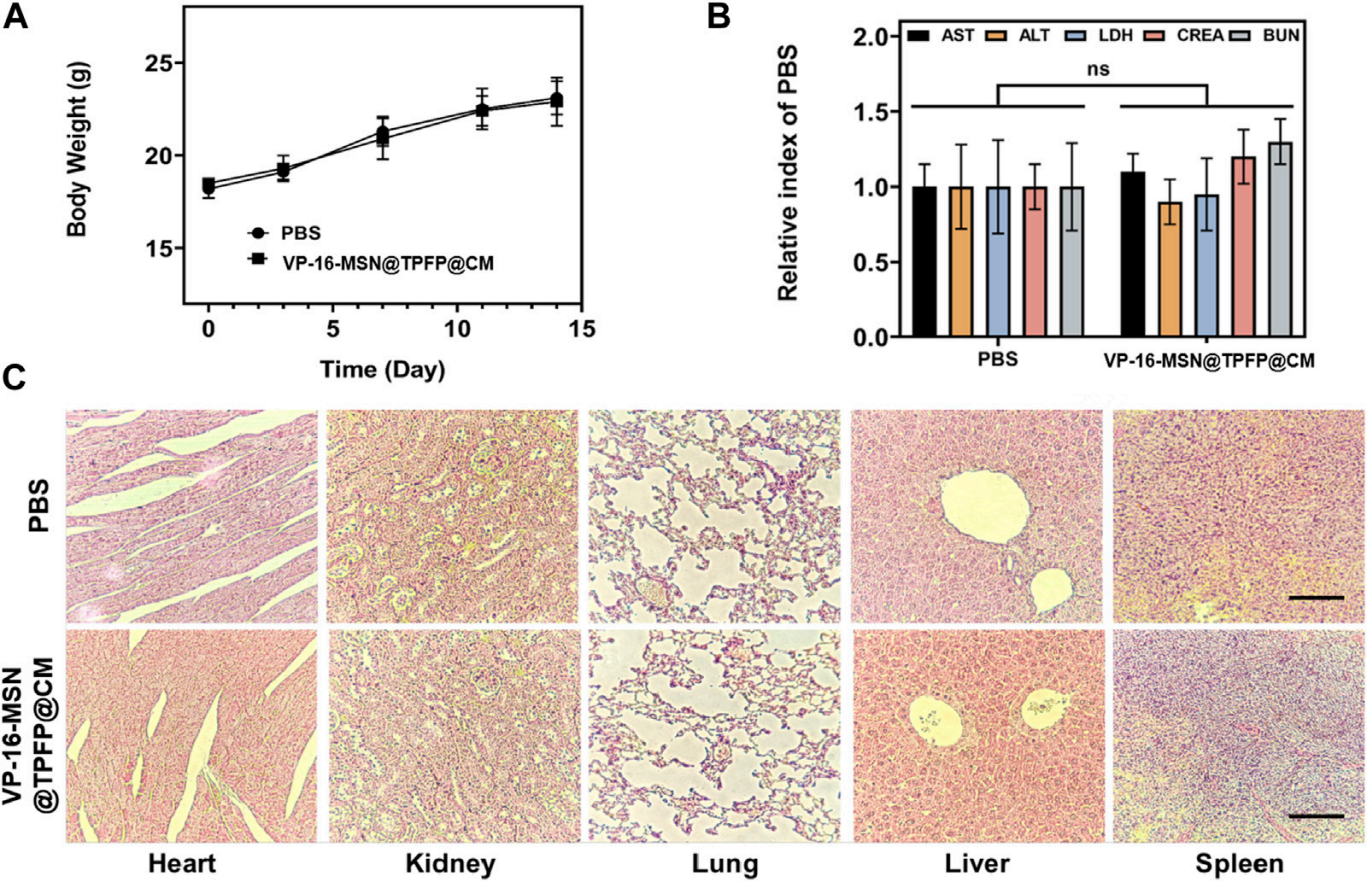
**(A)** Body weight and **(B)** biochemical parameters of 4T1 tumor-bearing mice 14 days after nanoprobe treatment. Data is represented as the mean ± SD (*n* = 3). **(C)** Histological evaluation from the major organs, including liver, spleen, kidney, heart, and lungs, of 4T1 tumor-bearing mice. Scale bars represent 50 µm.

Biosafety is a prerequisite for the clinical translation of biomaterials. Therefore, we examined the body weight, serum biochemical index, and histology of the major organs to evaluate the systemic toxicity of VP-16-MSN@TPFP@CM. No weight loss was observed in the VP-16-MSN@TPFP@CM group compared to the saline control group (Figure 5A). The levels of serum chemistry indices, including albumin (ALB), alanine transaminase (ALT), aspartate aminotransferase (AST), blood urea nitrogen (BUN), and Serum creatinine (CRE), did not change remarkably in any of the VP-16-MSN@TPFP@CM groups (Figure 5B). Furthermore, H&E staining of the liver, spleen, kidney, lung, and heart indicated the absence of pathological damage to the major organs during exposure (Figure 5C). Collectively, these results indicated that VP-16-MSN@TPFP@CM is biologically safe.

## 4 Conclusion

In summary, we developed an MSN-protected H_2_O_2_ imaging system that preserved the responsiveness of TPFP to ROS and achieved spatiotemporal synergy between chemotherapy and malignant prognosis prediction in breast cancer. Notably, VP-16-MSN@TPFP@CM was highly specific towards H_2_O_2_. Furthermore, VP-16-MSN@TPFP@CM exhibited a higher fluorescence enhancement than VP-16-MSN@TPFP *in vivo*, which was achieved by homologous cancer membrane cloaking with better tumor targeting and immune system evasion. Our results suggest that this probe could enable the evaluation of H_2_O_2_ pathology in chemotherapeutic cancer models and provide new insights into oxidative stress during chemotherapy. Given the recent translation of fluorescent imaging into early clinical trials and the high biocompatibility of the nanoprobe with further refinement, our approach paved the way for specific imaging of oxidative stress in solid tumors after treatment and provided a promising technology for precise prognostic predictions.

## Data Availability

The raw data supporting the conclusion of this article will be made available by the authors, without undue reservation.
